# The overexpression of salivary cytokeratins as potential diagnostic biomarkers in head and neck squamous cell carcinomas

**DOI:** 10.18632/oncotarget.19731

**Published:** 2017-07-31

**Authors:** Kai Dun Tang, Liz Kenny, Chris Perry, Ian Frazer, Chamindie Punyadeera

**Affiliations:** ^1^ The School of Biomedical Sciences, Institute of Health and Biomedical Innovation, Queensland University of Technology, Kelvin Grove, Queensland, Australia; ^2^ The Translational Research Institute, Woolloongabba, Australia; ^3^ School of Medicine, University of Queensland, Royal Brisbane and Women's Hospital, Brisbane, Queensland, Australia; ^4^ Central Integrated Regional Cancer Service, Queensland Health, Brisbane, Queensland, Australia; ^5^ Department of Otolaryngology, Princess Alexandra Hospital, Woolloongabba, Queensland, Australia; ^6^ The University of Queensland Diamantina Institute, Translational Research Institute, Woolloongabba, Queensland, Australia

**Keywords:** saliva, cytokeratin, head and neck squamous cell carcinoma, human papillomavirus

## Abstract

**Background:**

Cytokeratin (CK) intermediate filaments are demonstrated to have enormous potential in regulating cellular motility and cancer progression. There are more than 20 divergent CKs that have been identified, of which CK 8, 17, 18 and 19 are reported to be elevated in the tumour biopsies of head and neck cancer squamous cell carcinoma (HNSCC) patients. However, CK expression profiles in the saliva of HNSCC patients have not been investigated. We aim to investigate the mRNA expression profiles of CKs in saliva collected from healthy controls, HPV-negative and -positive HNSCC patients.

**Methods:**

Oral rinse samples were collected from 42 cancer-free healthy controls (age-matched) and patients who have been diagnosed with HPV-negative (n = 20) and -positive (n = 48) HNSCC.

**Results:**

Here, we report that the mRNA expression profiles of CKs differed in saliva collected from healthy controls and HNSCC patients. The mRNA expression levels of CK 8 and 18 were significantly elevated in saliva collected from HPV-negative HNSCC patients; whilst, CK 17 and 19 were expressed at a higher mRNA level in saliva collected from HPV-positive HNSCC patients compared to healthy controls. Importantly, receiver operating characteristic (ROC) analysis showed salivary CK 8 and 18 to have superior sensitivity and specificity in discriminating the HPV-negative HNSCC patients from healthy controls (80% and 86%) as well as between HPV-negative and -positive HNSCC patients (75% and 81%).

**Conclusion:**

In summary, we have demonstrated that an aberrant expression of salivary CKs may serve as a potential non-invasive diagnostic biomarker in HNSCC.

## INTRODUCTION

Head and neck cancer is a heterogeneous group of tumours arising from various anatomic structures including the nasal and oral cavity, oropharynx, larynx and hypopharynx [1]. More than 90% of these are head and neck squamous cell carcinomas (HNSCC) [2] and is the sixth most common cancer worldwide [3]. The incidence of HNSCC associated with traditional risk factors (tobacco use and alcohol consumption) is declining, whereas high-risk human papillomavirus (HPV)-positive HNSCC incidence is increasing in the western world, [4–8] including Australia [9].

Although the major risk factors for HNSCC are identified and have been used as prognostic biomarkers in the recent decades, disappointingly, there is no significant improvement in the overall survival rates of HNSCC (<50% survival in five years) compared to other cancer types like breast, colorectal and prostate [10–12]. This may be due to the high frequency of locoregional recurrences, limiting the development of new therapeutic strategies for HNSCC patients. Furthermore, most of these tumours are tiny in the early stages of tumour progression and are located in obscure regions of the head and neck anatomy, leading to misdiagnosis [13]. Therefore, saliva has been championed as the next generation body fluid by virtue of convenience sampling, non-invasive nature and serial sampling capabilities aiming at broader community-based screening [14–17].

Cytokeratins (CKs) are a family of cytoskeletal intermediate filament proteins that are commonly found in epithelial tissues [18]. CKs can be classified into two main groups according to their molecular weight and isoelectric point: Type I and Type II. Type I CKs (9 to 20) are more acidic and have a smaller molecular weight (40 - 64 kDa); whereas Type II CKs (1 to 8) are considered as neutral or basic and have a relatively large molecular weight (52 - 68 kDa) [19]. The expression of CKs can be sub-divided into categories depending on the degree of epithelial cells differentiation and maturation [20, 21]. For instance, cornified cells express CK 1, 2, 10 and 11; stratified cells express CK 4 and 13; basal cells express CK 5 and 14; hyperproliferative cells express CK 6 and 16 and simple cells express CK 7, 8, 18 and 19 [22–24]. As reported by previous studies, tissue polypeptide antigen (TPA), tissue polypeptide specific antigen (TPS), and CYFRA 21-1 are the most commonly used CK biomarker in various epithelial cell-associated carcinomas [25–27].

The aberrant expression of CKs has been reported to be associated with HNSCC tumour development and progression [28–30]. Indeed, these studies have only focused on investigating the diagnostic potential of CKs expression in both tumour biopsies and plasma, not in saliva. We hypothesized that the overexpression of CKs in saliva can be served as a potential diagnostic biomarker to discriminate healthy controls from HPV-negative and -positive patients. The aims of this study were two-fold: firstly, to investigate the mRNA expression profiles of CKs in saliva collected from healthy controls and HNSCC patients. Secondly, to determine the diagnostic potential of salivary CK mRNA expression levels in HNSCC.

## RESULTS

### Population characteristics

Healthy controls (n=42) and HNSCC patients (n=20 for HPV-negative HNSCC and n=48 for HPV-positive HNSCC) were recruited in this study (refer to Table 1). Most of the participants were male (70%) compared with females (30%). The mean age for healthy controls were 54 years (SD = 10; range = 33 – 72 years); HPV-negative HNSCC patients were 60 years (SD = 11; range = 20 – 92 years) and HPV-positive HNSCC patients were 63 years (SD = 13.2; range = 32 −87 years). The majority ethnicity in healthy controls, HPV-negative and -postive HNSCC patients were Caucasian, 92.9%, 95% and 98%, respectively. Healthy controls were more susceptible to be non-smokers than HPV-negative and -positive HNSCC patients. Healthy controls (42.9%) were classified as non-smokers, whilst 20% and 37.5% of HPV-negative and -positive HNSCC patients were non-smokers. About 80% of HPV-negative HNSCC patients were current and former smokers, whilst current and former smokers in the healthy controls and HPV-positive patients were 45% and 62% respectively.

**Table 1:** Participants demographic characteristics

		HNSCC Patients	
	Controls	HPV-negative	HPV-positive
	(n = 42)	(n = 20)	(n = 48)
**Gender**			
Male	29 (69.0)	16 (80.0)	46 (95.8)
Female	13 (31.0)	4 (20.0)	2 (4.2)
**Age (years)**			
<50	13 (30.9)	2 (10.0)	7 (14.6)
50 - 60	17 (40.5)	4 (20.0)	17 (35.4)
>60	12 (28.6)	14 (70.0)	24 (50.0)
**Race and ethnicity**			
Caucasian	39 (92.9)	19 (95.0)	47 (98.0)
Other	3 (7.1)	1 (5.0)	1 (2.0)
**Smoking status**			
Smokers	4 (9.5)	5 (25.0)	4 (8.3)
Ex-smoker	15 (35.7)	11 (55.0)	26 (54.2)
Non-smoker	18 (42.9)	4 (20.0)	18 (37.5)
N/A	5 (11.9)	0 (0)	0 (0)
**Tumour characteristics**			
AJCC TNM Stage I		1 (5.0)	2 (4.2)
Stage II		0 (0)	1 (2.1)
Stage III		6 (30.0)	5 (10.4)
Stage IVa		6 (30.0)	34 (70.8)
Stage IVb		3 (20.0)	5 (10.4)
Stage IVc		0 (0)	1 (2.1)
N/A		3 (15.0)	0 (0)
**Tumour anatomic sites**			
Oral cavity		5 (25.0)	4 (8.3)
Oropharynx		10 (50.0)	44 (91.7)
Larynx		1 (5.0)	0 (0)
Hypopharynx		3 (15.0)	0 (0)
Nasopahrynx		1 (5.0)	0 (0)
**Differentiation status**			
Well differentiated		0 (0)	0 (0)
Well to moderately differentiated		2 (10.0)	3 (6.3)
Moderately differentiated		10 (50.0)	9 (18.8)
Moderately to poorly differentiated		2 (10.0)	6 (12.5)
Poorly differentiated		3 (15.0)	10 (20.8)
N/A		3 (15.0)	20 (41.7)

According to the TNM classification system of AJCC for HPV-negative and -positive HNSCC patient staging was as follows: Stage I (5% and 4.2%), II (0% and 2.1%), III (30% and 10.4%), IVa (30% and 70.8%), IVb (20% and 10.4%) and IVc (0% and 2.1%) respectively. Most cases of HPV-negative and -positive HNSCC patients were diagnosed at an advanced stage IV (49 of 66; 72%). HPV-negative HNSCC cancer sites were mostly oropharyngeal and oral cavity (50% and 25%) whilst laryngeal, hypopharyngeal and nasopharyngeal were 5%, 15% and 5% respectively. Compared to HPV-negative HNSCC patients, the most common anatomical site found in HPV-positive HNSCC patients were oropharyngeal and oral cavity (91.7% and 8.3%). There were few cases of well differentiated squamous cell carcinoma found in both HPV-negative and -positive HNSCC patients. Most of the HPV-negative (75%) and -positive (50%) HNSCC patients were considered as moderate and poor squamous cell carcinoma.

### The mRNA expression levels of cytokeratin in saliva collected from healthy controls and HNSCC patients

**Figure 1:**
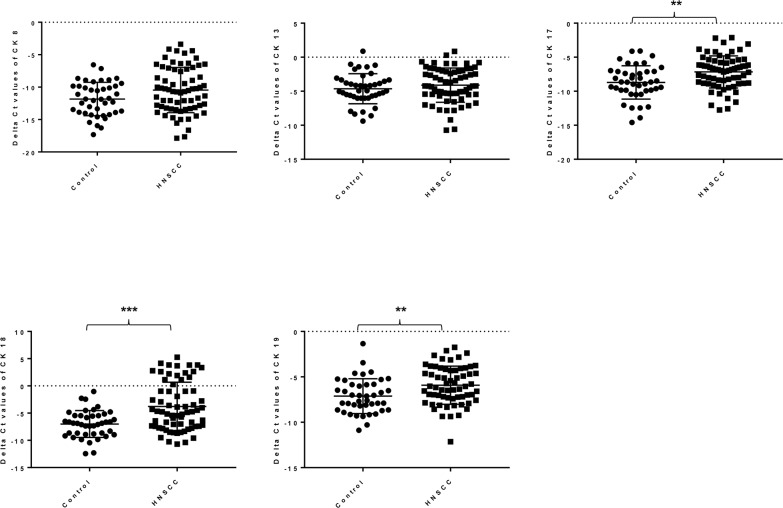
The mRNA relative expression level of CK 8, 13, 17, 18 and 19 in saliva collected from healthy controls (n=42) and HNSCC patients (n=68) using RT-qPCR Results were normalized to an internal control (beta-actin) and are presented as delta Ct values. Statistically significant differences (p < 0.05) were determined using Mann-Whitney U-test. (*p* values: ** < 0.005, *** < 0.001).

Although CK is one of the well-known tumour biomarkers for a number of tumour types including HNSCC, its expression level in saliva still remains to be elucidated. To address this question, we first investigated whether the saliva collected from healthy controls and HNSCC patients express the mRNA levels of CK 8, 13, 17, 18 and 19. As shown in Figure 1, the mRNA expression levels of CK 17, 18 and 19 was significantly upregulated in saliva collected from HNSCC patients compared to healthy controls, albeit CK 8 and 13. However, there was only a slightly upregulation of CK 8 mRNA expression level in the saliva collected from HNSCC patients compared to healthy control. Hierarchical clustering analysis revealed that the CKs mRNA expression patterns in saliva differed between healthy controls and HNSCC patients as shown in Supplementary Figure 1. These data strongly support the notion that tumour biopsies can potentially be replaced by saliva in the future.

### The overexpression of cytokeratin 8 and 18 mRNA levels in saliva collected from HPV-negative HNSCC patients

**Figure 2:**
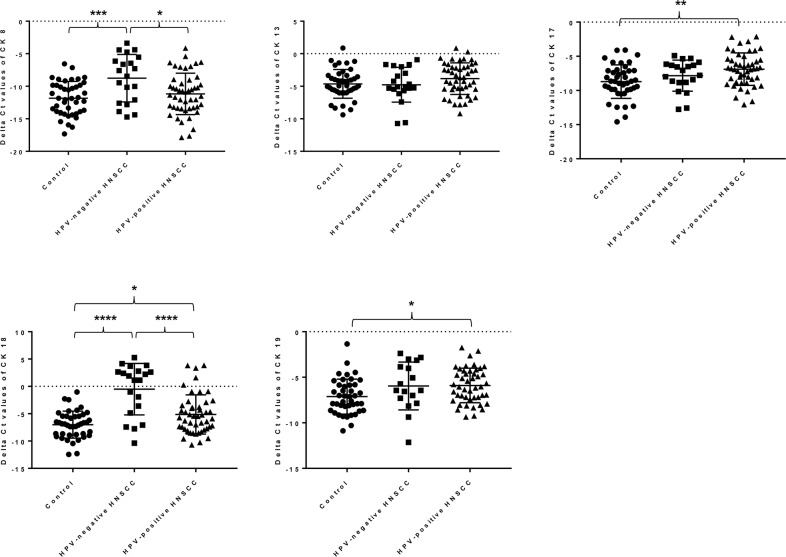
The mRNA relative expression level of CK 8, 13, 17, 18 and 19 in saliva collected from healthy controls (n=42), HPV-negative (n=20) and -positive HNSCC patients (n=48) by RT-qPCR Results were normalized with internal control and are presented as delta Ct values. Statistically significant differences (p < 0.05) among these three groups were determined using ordinary one way ANOVA. (*p* values: * < 0.05, ** < 0.005, *** < 0.001, **** < 0.0001).

**Figure 3:**
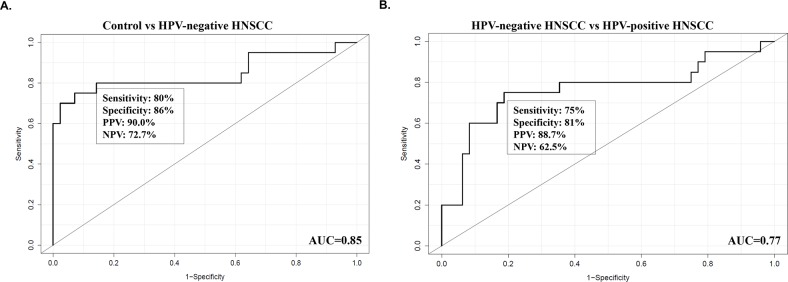
The receiver operator characteristic (ROC) curve analysis for the mRNA expression level of salivary CK 8 and 18 in healthy controls and HNSCC patients, indicating an area under the curve (AUC) with the diagnostic power to discriminate the HPV-negative HNSCC patients from healthy controls **(A)** as well as between HPV-negative and -positive HNSCC patients **(B)**.

Current literature reports that the expression of CKs is able to distinguish the HPV status in HNSCC patients [31] and hence, we hypothesized that its expression in saliva will potentially have similar expression trend as in tumor biopsies. As shown in Figure 2, the mRNA expression levels of CK 8 and 18 were significantly upregulated in saliva collected from HPV-negative HNSCC patients compared to healthy controls and HPV-positive HNSCC patients. Interestingly, no significant increase in both CK 8 mRNA levels was detectable in saliva collected from HPV-positive HNSCC patients when compared to healthy controls. Most importantly, using ROC curve analysis, the mRNA expression levels of CK 8 and 18 in saliva was able to distinguish the HPV-negative HNSCC patients from the healthy controls (Area under the curve (AUC) = 0.85, Sensitivity 80% and Specificity 86%, Positive predictive values (PPV) 90.0% and Negative predictive values (NPV) 72.7%) as well as between HPV-negative and -positive HNSCC patients (AUC = 0.77, Sensitivity 75% and Specificity 81%, PPV 88.7% and NPV 62.5%) as shown in Figure 3A and 3B. These findings further support the potential clinical utility of salivary CK 8 and 18 in differential diagnosis between HPV-negative and -positive HNSCC patients.

### The overexpression of cytokeratin 17 and 19 mRNA levels in saliva collected from HPV-positive HNSCC

Conversely, the mRNA expression levels of CK 17 and 19 were significantly elevated in saliva collected from HPV-positive patients compared to healthy controls as shown in Figure 2. However, the mRNA expression levels of CK 17 and 19 in saliva did not show any significant differences between healthy controls and HPV-negative HNSCC patients as well as HPV-negative and -positive HNSCC patients. Taken together, these results clearly highlight the potential diagnostic value of salivary CK 17 and 19 in discriminating the HPV status in HNSCC.

## DISCUSSION

The clinical diagnosis in HNSCC patients is challenging compared to other cancers as they are difficult to radiologically resolve due to the tumour size and anatomical region [13]. Therefore, diagnostic and predictive biomarkers are urgently needed to improve overall survival rates in HNSCC patients. Human saliva is a unique body fluid which has been widely used as non-invasive diagnostic markers for HNSCC [32–34]. Owing to its close proximity to the oral cavity, tumours may secrete and/or shed tumour-specific biomolecules directly into saliva. We are the first group to demonstrate that the expression profile of CKs in saliva collected from HNSCC patients is strongly correlated with blood and tissues expression levels from HNSCC patients. More importantly, the mRNA expression profile of CKs in saliva is able to discriminate the HPV-negative HNSCC from HPV-positive HNSCC.

Ample evidence has suggested that the CKs expression profile is associated with a range of different cancers including breast, colorectal, prostate and head and neck [30, 35–37]. Notably, CK 8, 17 and 18 were elevated in HNSCC tumour tissues compared to normal tissues [29, 38, 39]. The circulating fragments of CK 19 were significantly increased in the serum and saliva collected from HNSCC [40–42]. Similarly, in our saliva study, compared to healthy controls, HNSCC patients expressed a higher level of CK 17, 18 and 19 mRNA. Surprisingly, there was no significant difference between the CK 8 and 13 mRNA expression levels in saliva from HNSCC patients when compared to healthy controls.

Recent studies reported that the majority of HPV-positive HNSCC tumours have distinct phenotypes. This was confirmed by our immunohistochemistry (IHC) results that most of them have a non-keratinizing morphology with basal cell features [43, 44] (data not shown), clearly demonstrating the diagnostic potential of CKs to discriminate the HPV status in HNSCC. It is worth noting that there is an association between CKs and HPV infection, but also their underlying mechanism. In both *in vitro* and *in vivo* studies, high-risk type HPV-16 E1^E4 was found to interfere and collapse the structure of CK 8 and 18 networks in the cytoplasm [45]. Consistent with previous studies, the mRNA expression levels of CK 8 and 18 were downregulated in saliva samples collected from HPV-positive HNSCC patients compared with HPV-negative HNSCC patients.

Intriguingly, our study was in line with previous studies that observed the upregulation of CK 17 and 19 mRNA expression levels in saliva collected from HPV-positive HNSCC patients. According to Hobbs et al., CK 17 was significantly upregulated in HPV-16 mouse models compared to wild type, suggesting the possible association between HPV-16 infection and CK 17 expression [46]. Meanwhile, Favia *et al*. demonstrated for the first time that CK 19 induced the protein expression of HPV E7, an oncogene that plays a major role in promoting the carcinogenesis of HNSCC via the release of viral E7 mRNA from the translational block controlled by CK 7 [47]. Further evidence is supported by Santoro *et al*. which CK 19 suggested as a potential differential diagnostic marker to discriminate the HPV status in HNSCC [20].

Aside from being employed as a diagnostic biomarker, CKs are also found to harbour potential prognostic value in various epithelial cell-associated carcinomas [48–50]. Importantly, numerous studies have shown the significant correlation between CKs (especially 8, 18 and 19) and poor prognosis of HNSCC [50, 51], warranting further investigation. In summary, we have reported significantly elevated expression levels of CK 8 and 18 in saliva collected from HPV-negative patients, with simultaneously elevated expression levels of CK 17, 18 and 19 in saliva collected from HPV-positive HNSCC patients. A future longitudinal study is warranted to investigate the diagnostic and prognostic values of these markers.

## MATERIALS AND METHODS

### Study design

This study was approved by the University of Queensland Medical Ethical Institutional Board [HREC No: 2014000679 and 2014000862]; Queensland University of Technology [HREC No: 1400000617 and 1400000641] and by the Princess Alexandra Hospital Ethics Review Board [HREC Number: HREC/12/QPAH/381]. We have recruited (n=68) patients who have been diagnosed with HPV-negative and -positive HNSCC from the Princess Alexandra Hospital, Woolloongabba, Queensland, Australia. In addition, 42 cancer-free healthy controls (age-matched) were also recruited. Participants’ gave written informed consent, prior to obtaining samples. Clinical stages of HNSCC patients were classified according to the Tumour-Nodal-Metastasis (TNM) classification of malignant tumours of the American Joint Committee on Cancer (AJCC) and the HPV status was evaluated by a pathologist using standard routine diagnostic testing of p16INK4a immunohistochemistry (IHC) as described in our previous studies [33].

### Oral rinse samples collection and processing

Oral rinse samples were collected from participants as described previously [52]. Briefly, participants were asked to swish and gargle for 1-2 minutes with 10 ml 0.9% saline and subsequently expectorate into a 50 mL falcon tube. Samples were immediately kept on dry ice and transported back to the laboratory for processing. Samples were thawed and centrifuged at 1000 × g for 15 mins at 4°C. Cell pellets were resuspended in 800 μL of Qiazol (Qiagen, Germantown, MD, USA) for RNA isolation and stored at −80°C until further analysis.

### RNA isolation

Total RNA was isolated from oral exfoliated cell pellets using combined method of Qiazol-Chloroform (Qiagen) and RNeasy Mini Kit (Qiagen) as per manufacturer protocol. Briefly, 130 μL of chloroform was added to Qiazol containing oral exfoliated cells and centrifuged at maximum speed for 15 mins at 4°C. The resulting aqueous phase was transferred to a clean eppendorf tube with an equal volume of 70% ethanol. Then, transferred to RNeasy Mini spin columns and following the manufacturer's instructions.

### RT-qPCR analysis

RNA (200 ng) was used to synthesize cDNA using the iScript cDNA Synthesis Kit (Bio-Rad, Hercules, CA, USA) following the manufacturer's instructions. qRT-PCR was carried out with the QuantStudio™ 7 Flex Real-Time PCR System (Applied Biosystems, Foster City, CA, USA). Sense and anti-sense primers targeted against the genes of interest are listed in Supplementary Table 1. The mRNA transcript level of human beta-actin was used as a normalizer.

### Statistical analysis

The mRNA expression levels of individual CKs in saliva between two cohorts (healthy controls and HNSCC patients) were compared using non-parametric analysis using Mann-Whitney U-test; while among the three cohorts (healthy controls, HPV-negative and -positive HNSCC patients) were compared using the ordinary one way ANOVA. P-values less than 0.05 were considered significant. A receiver operating characteristic (ROC) curve was used to discriminate healthy controls from HPV-negative HNSCC patients as well as between HPV-negative and -positive HNSCC patients using the salivary CKs mRNA expression levels. All the statistical analysis was performed using GraphPad Prism 7 software version 7 (GraphPad Software Inc., USA) and R Programming.

The hierarchical clustering analysis was determined using Morpheus (https://software.broadinstitute.org/morpheus).

## SUPPLEMENTARY MATERIALS FIGURES AND TABLES


