# Supplementary material for: The overexpression of salivary cytokeratins as potential diagnostic biomarkers in head and neck squamous cell carcinomas
Source: Oncotarget. 2017 Jul 31;8(42):72272–80. doi: 10.18632/oncotarget.19731 (PMC5641129; doi:10.18632/oncotarget.19731)
Supplement: Supplementary file 1 [file oncotarget-08-72272-s001.pdf]

The overexpression of salivary cytokeratins as potential diagnostic biomarkers in head and neck squamous cell carcinomas

SUPPLEMENTARY MATERIALS

REFERENCES

1. Makino T, Yamasaki M, Takeno A, Shirakawa M, Miyata H, Takiguchi S, Nakajima K, Fujiwara Y, Nishida T, Matsuura N, Mori M, Doki Y. Cytokeratins 18 and 8 are poor prognostic markers in patients with squamous cell carcinoma of the oesophagus. Br J Cancer. 2009; 101:1298-1306.
2. Ramirez-Miranda A, Nakatsu MN, Zarei-Ghanavati S, Nguyen CV, Deng SX. Keratin 13 is a more specific marker of conjunctival epithelium than keratin 19. Mol Vis. 2011; 17:1652-1661.
3. Zhang W, Dang E, Shi X, Jin L, Feng Z, Hu L, Wu Y, Wang G. The pro-inflammatory cytokine IL-22 up-regulates keratin 17 expression in keratinocytes via STAT3 and ERK1/2. PLoS One. 2012; 7:e40797.
4. Dimmler A, Gerhards R, Betz C, Gunther K, Reingruber B, Horbach T, Baumann I, Kirchner T, Hohenberger W, Papadopoulos T. Transcription of cytokeratins 8, 18, and 19 in bone marrow and limited expression of cytokeratins 7 and 20 by carcinoma cells: inherent limitations for RT-PCR in the detection of isolated tumor cells. Lab Invest. 2001; 81:1351-1361.
5. Mahajan K, Lawrence HR, Lawrence NJ, Mahajan NP. ACK1 tyrosine kinase interacts with histone demethylase KDM3A to regulate the mammary tumor oncogene HOXA1. J Biol Chem. 2014; 289:28179-28191.

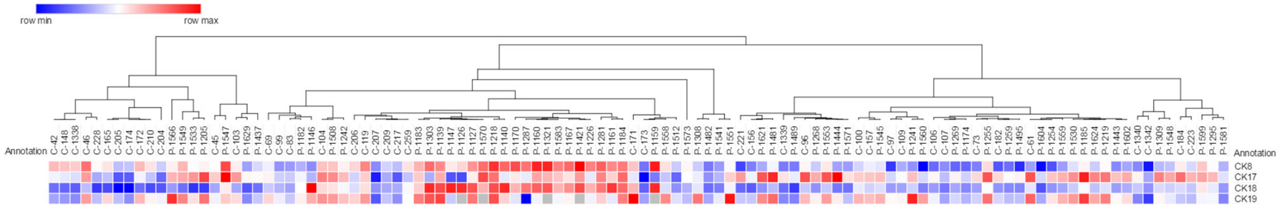

Supplementary Figure 1: The hierarchical clustering analysis was applied to stratify the mRNA expression pattern of CKs (8, 17, 18 and 19) in saliva collected from both healthy controls and HNSCC patients.

Supplementary Table 1: Primer sequence

Gene	Primer sequence	Reference
CK 8 F	5’-TAGCACTGGGAACAGGAGA-3’	[1]
CK 8 R	5’-TTTGACATTGGCAGAGCTA-3’	
CK 13 F	5’-CTGAACAAGGAGGTGTCTACCA-3’	[2]
CK 13 R	5’-ATAGCGGCACTCCGTCTCT-3’	
CK 17 F	5’-ACCATGCAGGCCTTGGAGA-3’	[3]
CK 17 R	5’-GTCTTCACATCCAGCAGGA-3’	
CK 18 F	5’-GAGACGTACAGTCCAGTCCTTGG-3’	[4]
CK 18 R	5’-CCACCTCCCTCAGGCTGTT-3’	
CK 19 F	5’-TGAGTGACATGCGAAGCCAATAT-3’	[4]
CK 19 R	5’-GCGACCTCCCGGTTCAAT-3’	
Beta-actin F	5’-CACCATTGGCAATGAGCGGTTC-3’	[5]
Beta-actin R	5’-AGGTCTTTGCGGATGTCCACGT-3’	

***CK = Cytokeratin
